# Phosphorus‐Graphene Nanosheet Hybrids as Lithium‐Ion Anode with Exceptional High‐Temperature Cycling Stability

**DOI:** 10.1002/advs.201400020

**Published:** 2015-01-28

**Authors:** Zhaoxin Yu, Jiangxuan Song, Mikhail L. Gordin, Ran Yi, Duihai Tang, Donghai Wang

**Affiliations:** ^1^ Department of Mechanical and Nuclear EngineeringThe Pennsylvania State UniversityUniversity ParkPA16802USA

**Keywords:** graphene, phosphorus, anode, lithium‐ion battery, high temperature, cycling stability

## Abstract

**A red phosphorus‐graphene nanosheet hybrid** is reported as an anode material for lithium‐ion batteries. Graphene nanosheets form a sea‐like, highly electronically conductive matrix, where the island‐like phosphorus particles are dispersed. Benefiting from this structure and properties of phosphorus, the hybrid delivers high initial capacity and exhibits promising retention at 60 °C.

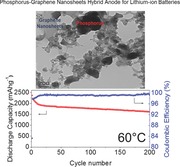

Lithium‐alloy anode materials for lithium‐ion batteries (LIBs), such as Si,^1^ Sn,^2^ and Ge,^3^ have attracted great attention in recent years due to their high theoretical capacity. The main challenge for the practical application of these materials is their large volume change (up to 300%) during lithium insertion and extraction.[[qv: 1c]],^4^ This volume change can cause critical issues including pulverization of the active material, unstable growth of the solid‐electrolyte interphase (SEI), and loss of contact between the active material and the conductive matrix, leading to poor capacity, low coulombic efficiency, and fast capacity fading.^5^ The situation becomes even worse when making it for practical application that necessitates the operation at high temperature (>50 °C).[[qv: 5b]],^6^

Recently, phosphorus (P) was found to electrochemically lithiate and form Li_3_P when used as a Li‐ion battery anode.^7^ This three‐electron‐transfer reaction of phosphorus with lithium provides an extremely high theoretical capacity of 2595 mAhg^−1^, which is almost seven times higher than that of graphite. Phosphorus has three main allotropes: white, black and red. White phosphorus is poison and chemically not stable, which cannot be used as anode materials. Black phosphorus is thermodynamically and chemically most stable one and has higher conductivity than red phosphorus. However, its synthesis is difficult and requires high temperature and high pressure, which leads to the lowest commercial value among the three forms. Different from white and black phosphorus, red phosphorus is chemically stable, abundant, low‐cost and eco‐friendly, which make it promising for high‐energy LIBs. Despite these advantages, similar to other intermetallic alloy, two intrinsic issues of red phosphorus are the main hurdles for its application for LIBs. Firstly, it has a very low electronic conductivity (1 × 10^−10^ S cm^−1^), which makes electrochemical redox reactions difficult.^8^ Secondly, its large volume expansion of up to 300% causes issues like those identified above for the more heavily‐studied alloy anodes.[[qv: 1c]]

Various carbon materials have been utilized to modify phosphorus and enhance the overall conductivity of the electrodes. These phosphorus/carbon composites, including P/carbon black (7:3),^9^ P/graphite (1:3),^10^ and P/porous carbon (3:7;^11^ 1:1^12^) can significantly improve the rate and cycling stability of the phosphorus based anode. In the recently reported graphite/phosphorus composite, it was found that P‐C bonds were formed during ball‐milling; these are believed to play an important role in maintaining the contact of phosphorus and conductive graphite.^13^ In addition, highly conducive metals were also employed to form alloy with phosphorus to enhance the conductivity.^14^ For example, Sn and phosphorus with the molar ratio of 4:3 can form compound Sn_4_P_3_ via high‐energy ball‐milling, which delivers a capacity of 500 ∼ 700 mAhg^−1^.[[qv: 14a,d,15]] Despite this encouraging progress, the current research on phosphorus‐based Li‐ion anodes is still limited in the following ways. First, either too much inactive additive was introduced to these phosphorus‐based hybrids, such as P/carbon black (30 wt% carbon black), or the content of the low‐capacity additives in the hybrid is too high, such as in the P/graphite hybrid (75 wt% graphite) and P‐Sn alloy (58 wt% Sn). This dramatically reduces the capacity and thereby the energy density of whole electrode. Second, most publications focus on the electrochemical performance at room temperature, while the study of cells operated at aggressive conditions, such as at high temperature (>50 °C) is rare to date, which is necessitated for the real application. Third, only limited cycling (≤100 cycles) has been reported, which is far from the practical application that needs longer cycles.

Herein, we report a red phosphorus‐graphene nanosheet (P‐G) hybrid as anode for lithium‐ion batteries, prepared via ball milling low‐cost commercial red phosphorus and graphene stacks. The graphene stacks are exfoliated into nanosheets and hybridize with phosphorus particles to form a 3D integrated network, which significantly improves the conductivity^16^ and prevents loss of contact between P/Li_3_P and graphene during the lithiation/delithiation process. Moreover, P‐O‐C bond is found in the hybrid material, and helps to maintain contact between the phosphorus and graphene. This structure and inter‐component bonding enable the P‐G hybrid anode to achieve a high initial capacity of 2517 mAhg^−1^ at room temperature, with 60% capacity retention after 300 cycles at 0.1 C (1C = 2600 mAhg^−1^) rate and good rate performance. At the elevated temperature of 60 °C, performance of the hybrid anode is also found to be excellent, showing 74% capacity retention after 200 cycles at a higher rate of 0.2 C.

The synthesis process of the P‐G hybrid is schematically summarized in **Figure** **1**. Commercially available micro‐sized red phosphorus was ball‐milled with graphene stacks to obtain the final product: phosphorus‐graphene nanosheet (see experimental section for details). It is well known that ball milling can grind large particles down to smaller micron‐sized or even nano‐sized particles, and layered materials can be exfoliated by shear stress during grinding. The bulk red phosphorus was thus ground to particles with much smaller size, shortening the lithium‐ion transport path and mitigating particle fracture caused by volume change of phosphorus during lithiation and delithiation.[[qv: 5a]],^17^ Graphene stacks were exfoliated into flexible graphene nanosheets (Figure S1, Supporting Information), and contact with each other to form highly conductive matrix.^18^

**Figure 1:**
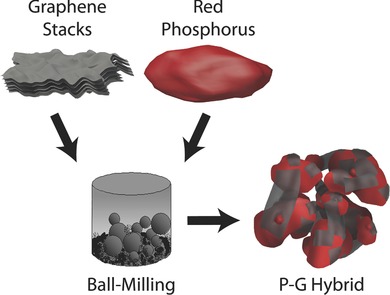
The preparation process from red phosphorus and graphene stacks to the P‐G hybrid.

Raw red phosphorus, graphene stacks, a mixture of phosphorus and graphene stacks (P/G mixture) and the ball‐milled P‐G hybrid were investigated by the X‐ray diffraction (XRD) technique (Figure S2, Supporting Information). The starting red phosphorus powder has a medium‐range ordered structure based on its three broad diffraction peaks located at 2θ values of 15.0°, 31.6°, and 45.4°.^7^, ^19^ The peak at a 2θ of 26.4° in the pattern of graphene stacks can be assigned to hexagonal crystalline graphite (JCPDS No. 41–1487). All the diffraction peaks corresponding to red phosphorus and graphene stacks are visible in the diffraction pattern of the P/G mixture. After ball milling, appearance of broad peaks in the XRD pattern of P‐G hybrid, rather than sharp ones in the P/G mixture, suggests that the resulting product is an amorphous red phosphorus‐graphene nanosheets hybrid. The halo‐like electron diffraction pattern of P‐G hybrid (**Figure** **2**d) further demonstrates the amorphous state.

**Figure 2:**
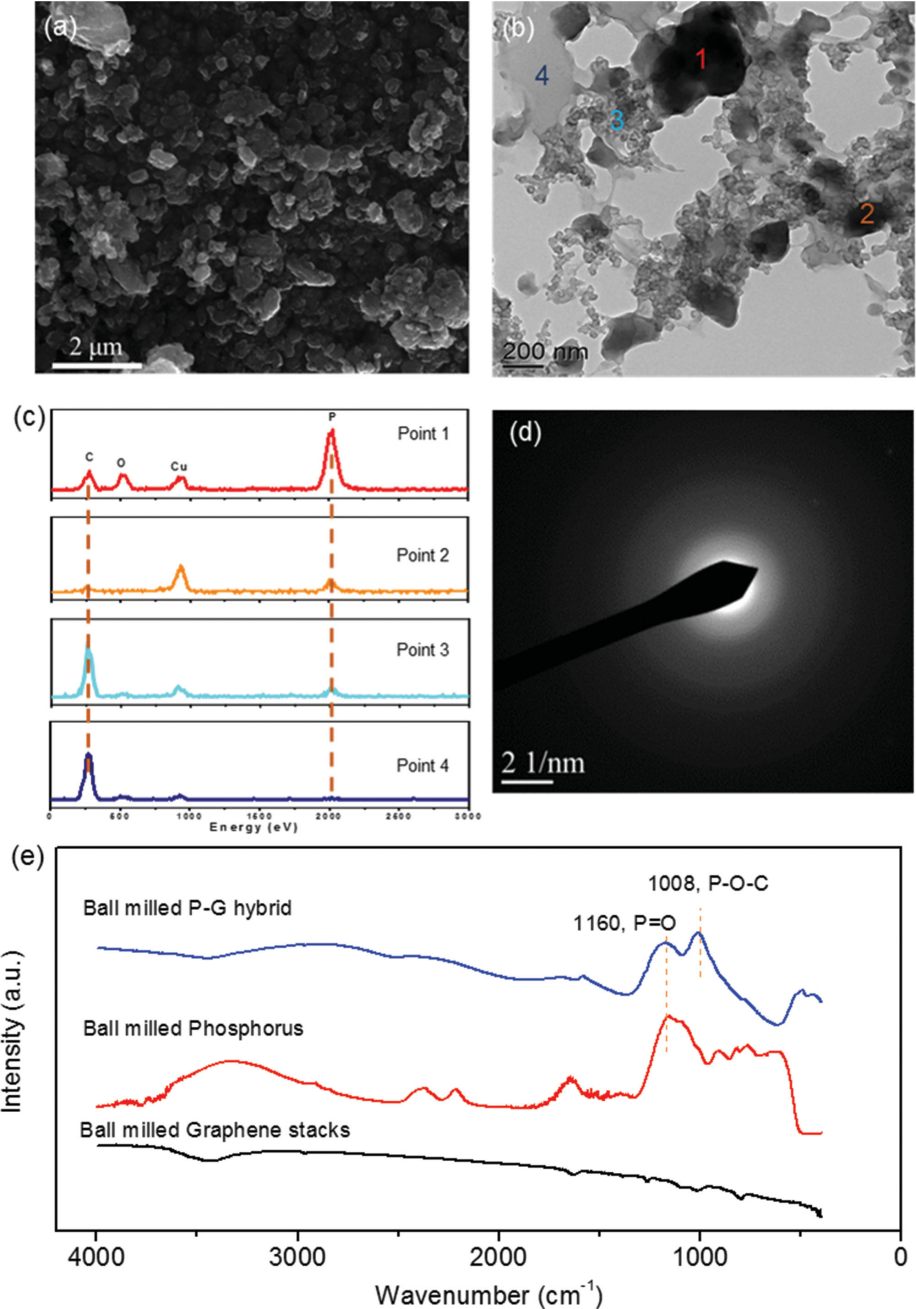
a) SEM image of the P‐G hybrid; b) TEM image of P‐G hybrid; c) EDS spectra corresponding to points 1–4 in (b); d) electron diffraction pattern of point 1 in (b); e) FTIR spectra of ball milled P, ball milled G and P‐G hybrid.

The morphology of P‐G hybrid was investigated by electron microscopy. A transmission electron microscopy (TEM) image of graphene stacks and a scanning electron microscopy (SEM) image of raw phosphorus before ball‐milling are presented in Figure S3a and S3b, Supporting Information, respectively. It is clearly found that graphene stacks have multilayer stacked structure with a micro‐sized plane, and the raw phosphorus particles are above 50 μm in size. An SEM image of the ball‐milled P‐G hybrid is shown in Figure 2a, illustrating that much smaller particles are formed after balling milling. A TEM image and the corresponding energy‐dispersive X‐ray spectroscopy (EDS) spectra of the P‐G hybrid are shown in Figure 2b and 2c, respectively. From the EDS, the ratio of carbon to phosphorus keeps increasing from point 1 to point 4, indicating that the light region (point 4) in Figure 2b is carbon rich and the dark island‐like region (point 1 and 2) is phosphorus rich (Cu signal is from grid). The graphene nanosheets, which the carbon is derived from, contact one another to form a sea‐like highly‐electronically‐conductive matrix. The island‐like phosphorus is dispersed in the conductive matrix and surrounded by graphene nanosheets. The TEM results indicate that phosphorus and graphene nanosheets form a 3D integrated structure in the hybrid during the ball milling process.

Fourier transform infrared spectroscopy (FTIR) was used to investigate the potential of the chemical bonding between phosphorus and graphene nanosheets, as shown in Figure 2e. The peaks of 1160 cm^−1^ and 1080 cm^−1^ in raw phosphorus sample are assigned to the P = O and P‐O‐P bonds, respectively, due to the presence of oxide on the commercial phosphorous particles.^19^ After ball‐milling, the intensity of these peaks decreases, while a new peak appears at 1008 cm^−1^; this peak is assigned to P‐O‐C asymmetric stretching.[[qv: 2a]] This chemical bonding strongly binds the phosphorus to the graphene, promoting intimate P‐G contact and increasing the durability of the structure.

In order to investigate the electrochemical properties of the P‐G hybrid, CR2016‐coin cells were made in an argon‐filled glove box with the P‐G hybrid as the working electrode and lithium foil as the counter electrode. All cells were tested between 0.01 V and 2.0 V vs. Li/Li^+^. Cyclic voltammetry (CV) of the P‐G hybrid is shown in **Figure** **3**a. A clear peak at ca. 0.25 V was only observed during the first lithiation step, which is believed to be an activation process that lithium ion inserting into polymeric phosphorus.[[qv: 1b]] After the first cycle, three peaks (I, II and III) are found in the cathodic scan. The first two peaks from 1.5 V to 0.2 V corresponds to the continuous lithiation process to form Li_x_P (x=1–3) intermediates. The third peak, appearing between 0.2 V and 0.01 V, is believed to originate from lithium intercalation into graphene nanosheets.^20^ In the anodic scan, peak IV, centered at 0.2 V, corresponds to lithium ion de‐intercalation from graphene nanosheets, as shown in the inset of Figure 3a. Peaks V (1.05 V), VI (1.26 V), and VII (1.68 V) are possibly due to a stepwise delithiation process from the discharged Li_x_P phase to Li_y_P (1 ≤ y < x ≤ 3) intermediates and finally to P.^7^

**Figure 3:**
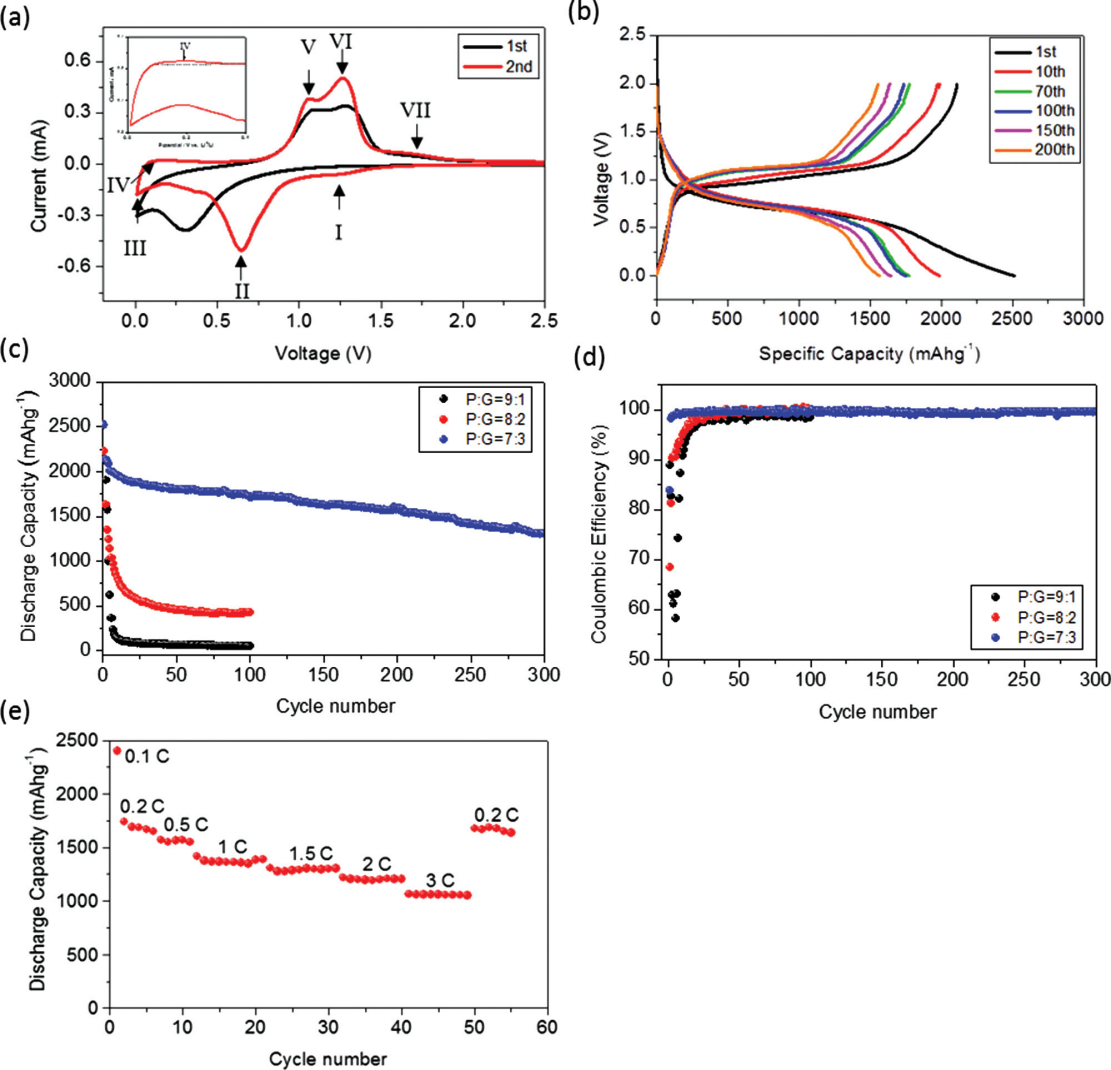
a) Cyclic voltammetry of a P‐G hybrid (70% P) cell at a scan rate of 0.1 mVs^−1^; b) voltage profiles of a P‐G hybrid (70% P) cell at different cycle numbers; c) cycling stability and d) Coulombic efficiency of P‐G hybrid (different composition) cells at a current density of 130 mAg^–1^ for the 1^st^ cycle and 260 mAg^−1^ for the subsequent cycles; and e) rate performance of a P‐G hybrid (70% P) cell at C‐rate between 0.1C and 3C (1C = 2600 mAg^−1^).

The voltage profiles of P‐G hybrid from 1^st^ cycle to 200^th^ cycle are illustrated in Figure 3b. During the first cycle, the initial discharge and charge capacities of the P‐G hybrid electrode are 2517 and 2110 mAhg^−1^ based on the mass of P, respectively, giving an initial coulombic efficiency of ca. 84%, corresponding to 96.7% phosphorus utilization compared with the theoretical specific capacity of phosphorus. The irreversible capacity of the first cycle is mainly attributed to the formation of an SEI layer on the electrode surface[[qv: 1b]],^21^ and irreversible Li insertion into the graphene matrix,^22^ which is also observed for the first cycle of control electrodes using graphene nanosheets as the active material (Figure S4, Supporting Information). The cycling performance and coulombic efficiency of the P‐G hybrid at room temperature are displayed in Figure 3c and d, respectively, where the effect of different composition of P‐G hybrid on the electrochemical performance is also studied. The cells were tested at a rate of 0.05 C for activation for the first cycle and 0.1 C for the following cycles. From Figure 3c, with 10 wt% graphene nanosheets, the P‐G hybrid shows very fast capacity fading, while it is significantly improved with the increase of the graphene content to 20%. Especially when the graphene nanosheets content increases to 30%, the P‐G hybrid shows a high specific capacity and good cycling stability. The trend, the higher content of graphene nanosheets the better electrochemical performance, indicates that sufficient graphene nanosheets are required to form a sea‐like highly electronically conductive matrix. Further increasing the graphene nanosheet content is not recommended, which will decrease the overall capacity due to the lower capacity of graphene nanosheets. After 300 cycles, the P‐G hybrid (70% P) still delivered 1283 mAhg^−1^ based on the mass of P (898 mAhg^−1^ based on P‐G), having retained 60% of its capacity with respect to the second cycle capacity of 2137 mAhg^−1^ based on P. As graphene stacks were exfoliated into graphene nanosheets, the stable cycling capacity of the graphene nanosheet is expected to be low^23^ and thus neglected for calculating specific capacity of phosphorus. The efficiency quickly reached 99.0% after 6 cycles and stayed above 99.0% in the following cycles. The rate performance of the P‐G hybrid was also evaluated using rates between 0.1 C to 3 C, as shown in Figure 3e. The P‐G hybrid showed excellent rate performance; even at 3 C, it still delivered a high reversible capacity of ca. 1080 mAhg^−1^ based on P (756 mAhg^−1^ based on P‐G). When the rate was restored to 0.2 C after 50 cycles, the specific capacity returned to 1676 mAhg^−1^ based on P (1173 mAhg^−1^ based on P‐G), which is close to the 1691 mAhg^−1^ based on P of 3^rd^ cycle. This indicates that the electrode cycling was reversible even at high current densities.

In order to understand the reason for the superior cyclability and rate capability of the P‐G hybrid, the energy‐filtered TEM (EFTEM) and the corresponding element mapping of P‐G hybrid after 150 cycles were detected and shown in **Figure** **4**a–c. The P‐G hybrid still maintained the structure after long‐term cycling without noticeable disconnection, indicating the excellent durability of the structure. The mixture of ball‐milled phosphorus and ball‐milled graphene stacks (without chemical bond between phosphorus and carbon, Figure S5, Supporting Information) was further tested as control sample (P/G control sample) under the same conditions to investigate the role of P‐O‐C bond in keeping the structure integrity. The fast capacity decay of P/G control sample demonstrates the important contribution of P‐O‐C bond in maintaining the electrical connection between phosphorus and conductive matrix. Besides these reasons, the 3D integrated network enabled by graphene nanosheets is also considered to contribute to the good cycling retention, which will be further discussed in the high temperature performance evaluation of P‐G hybrids and comparison of P‐CB hybrids below.

**Figure 4:**
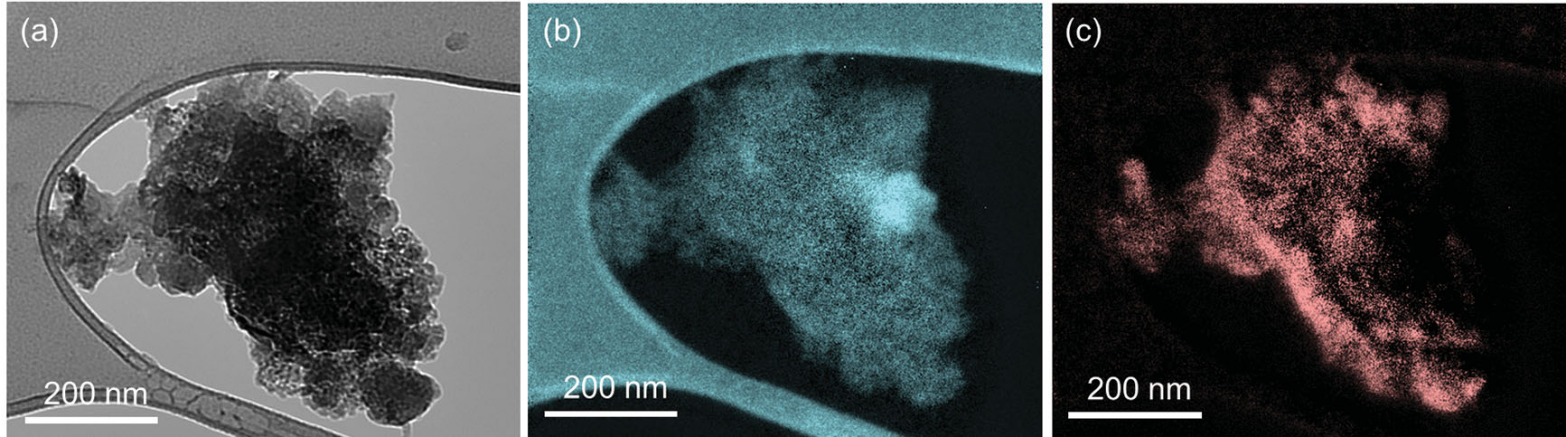
a) TEM images of the P‐G hybrid electrode after 150 cycles; the corresponding elemental mapping of b) carbon and c) phosphorus.

The operational temperature range of lithium‐ion battery is dictated by its specific application. The LIBs for consumer electronics work within the temperature window from –20 °C to 60 °C. For hybrid electrical vehicle, batteries need to work in even wider window (–30 °C to 80 °C). Therefore, the electrochemical performance of lithium‐ion battery at elevated temperature (>50 °C) is of great importance for real application. Even though many groups report the electrochemical properties of phosphorus at room temperature, their behavior at elevated temperature (>50 °C) is rarely studied up to now. In order to investigate the high temperature electrochemical properties, cells were tested at 0.1 C rate at room temperature for activation for the 1^st^ cycle and at 0.2 C at elevated temperature (60 °C) for the following cycles. Ball‐milled phosphorus‐carbon black (P‐CB) hybrid was used as a control sample. The electrochemical properties of the P‐G and P‐CB hybrid at 60 °C are illustrated in **Figure** **5**. Similar charge/discharge curves are detected for P‐G hybrid at 60 °C (Figure 5a), indicating the same electrochemical mechanism at high temperature as that at room temperature. P‐CB also shows similar voltage profiles (Figure 5b), demonstrating that it follows the same electrochemical process as the P‐G hybrid. Figure 5c and d show the cycling performance and coulombic efficiency of P‐G and P‐CB hybrid at 60 °C. The P‐G hybrid delivered initial capacity of ca. 2360 mAhg^−1^ based on the mass of P, with high initial columbic efficiency of ca. 83.0%. After 200 cycles, P‐G hybrid at elevated temperature delivered capacity of 1623 mAhg^−1^ based on P, 1136 mAhg^−1^ based on P‐G, with a retention of 73% with respect to the second cycle capacity of 2222 mAhg^−1^ based on P. The coulombic efficiency of P‐G hybrid at 60 °C (Figure 5d) increased rapidly up to 99.0% after 15 cycles with an average value of 99.2% within 200 cycles. Compared to P‐G hybrid, the P‐CB only shows the retention of 63% after 200 cycles, with respect to the second cycle capacity of 2076 mAhg^−1^ based on P.

**Figure 5:**
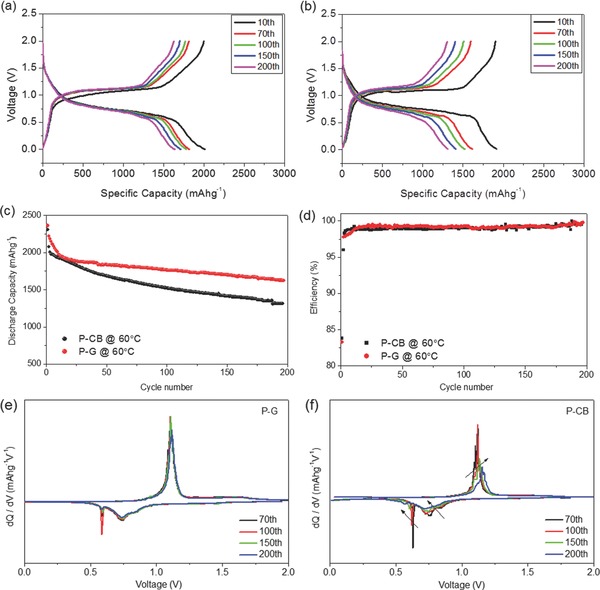
Voltage profiles of a) P‐G hybrid (70% P) and b) P‐CB hybrid (70% P) cells at different cycle numbers; c) cycling stability and d) coulombic efficiency of P‐G (70% P) and P‐CB (70% P) hybrid cells at a current density of 260 mAg^–1^ for the 1^st^ cycle and 520 mAg^−1^ for the subsequent cycles; and differential capacity (dQ/dV) curves of e) P‐G hybrid (70% P) and f) P‐CB hybrid (70% P) cells; for these tests, the first cycle was always performed at room temperature, while following cycles were at elevated temperature (60 °C).

Differential capacity (dQ/dV) plots of the charge/discharge profiles of P‐G and P‐CB are shown in Figure 5e and f, respectively. With increasing cycling numbers, curves in Figure 5e remain almost the same, suggesting the good contact between phosphors and conductive matrix and great cycling stability. In contrast, cathodic peaks in dQ/dV curves (Figure 5f) of P‐CB sample eventually shift to lower voltage region and anodic peaks to higher voltage region, accompanied with peak intensity decline, showing the increased polarization and fast capacity decay. These differences are believed to be originated from 3D integrated structure built up by graphene nanosheets and phosphorus, which ensures the good electrical contact and prevents the contact loss between phosphorus and graphene during cycling, leading to high cycle retention.

In summary, we successfully developed a phosphorus‐graphene nanosheet hybrid via simple ball milling low‐cost red phosphorus and graphene stacks. The graphene stacks are exfoliated into two‐dimensional large pieces of graphene nanosheets, which build up highly‐electronically‐conductive matrix. Large phosphorus particles are grounded to much smaller size and hybridize with graphene nanosheets to form a 3D integrated network. In addition, the content of graphene nanosheets has been optimized. Sufficient graphene nanosheets are required to build up 3D integrated network. Moreover, P‐O‐C chemical bonds, found between phosphorus and carbon in the hybrid, ensure an intrinsic electrical connection and a robust structure. As a result, high gravimetric specific capacity of 2362 mAhg^−1^ based on mass of P and excellent capacity retention within 300 cycles is achieved at room temperature. Even at elevated temperature of 60 °C, ca. 1623 mAhg^−1^ based on P with a retention of 73% after 200 cycles and high coulombic efficiency of 99.0% is achieved. Taking the facile and general method as well as the low cost starting materials into account, this P‐G hybrid is believed to be promising anode candidate for the next generation lithium‐ion battery.

## Experimental Section

*Preparation of P‐G Hybrid, P‐CB Hybrid, P/G Mixture, and P/G Control Sample*: The P‐G hybrid was prepared though a straightforward, industry‐accepted ball‐milling approach. The raw materials, commercial red phosphorus (Afla Aesar, 99%) and graphene stacks (XG Science, XG‐300) were mixed together with a mass ratio of 7:3. The mixture was then ball‐milled for 1000 min at a speed of 400 rpm in a stainless steel jar under argon atmosphere. P‐CB hybrid was prepared following the same procedure using carbon black as a carbon source. The P/G mixture used for XRD test was prepared by directly mixing raw red phosphorus and raw graphene stacks. The P/G control sample for electrochemical test was prepared by hand milling the ball milled phosphorus and ball‐milled graphene stacks with the ratio of 7:3. The phosphorus and graphene stacks were separately ball‐milled for 1000 min at a speed of 400 rpm under argon atmosphere before hand‐mill mixing.

*Characterization*: Powder XRD was collected on a Rigaku Miniflex II spectrometer with Cu K_α_ radiation. The morphology of the P‐G hybrid was investigated with scanning electron microscope (Nano630 FE‐SEM). TEM and EFTEM (JEOL 2010 LaB_6_) were used for microstructure investigation and elemental mapping. FTIR spectroscopy was performed as diffuse reflectance measurements with powder samples using a Bruker IFS 66/S FT‐IR spectrometer and Spectra‐Tech Collector II DRIFTS accessory; samples were diluted with KBr in approximately a 30:1 mass ratio.

*Electrochemical Measurement*: Electrochemical performance was tested using 2016‐type coin cells, which were assembled in an argon‐filled dry glove box (MBraun, Inc.). The working electrodes were prepared by casting a slurry composed of 70 wt.% active material, 15 wt.% Super P carbon black, and 15 wt.% sodium carboxymethylcellulose (NaCMC) binder (2 wt.% in water) on copper foil, drying at 100 °C under vacuum overnight, and punching out disks 12 mm in diameter. The mass loading of electrodes was 0.8–1.5 mg cm^−2^. 1M LiPF_6_ in a mixture of ethylene carbonate (EC), diethyl carbonate (DEC) and fluoroethylene carbonate (FEC) (1:1:0.2 by volume). Galvanostatic charge/discharge tests were carried out on a LAND battery tester between 0.01 V and 2.0 V versus Li/Li^+^. Cyclic voltammetry (CV) was performed at a scan rate of 0.1 mVs^−1^ within the range of 0.01–2.0 V using a CHI 660D electrochemical workstation.

## Supporting information

As a service to our authors and readers, this journal provides supporting information supplied by the authors. Such materials are peer reviewed and may be re‐organized for online delivery, but are not copy‐edited or typeset. Technical support issues arising from supporting information (other than missing files) should be addressed to the authors.

Supplementary
