# Supplementary material for: Phosphorus‐Graphene Nanosheet Hybrids as Lithium‐Ion Anode with Exceptional High‐Temperature Cycling Stability
Source: Adv Sci (Weinh). 2015 Jan 28;2(1-2):1400020. doi: 10.1002/advs.201400020 (PMC5115279; doi:10.1002/advs.201400020)
Supplement: Supplementary file 1 — Supplementary [file ADVS-2-0c-s001.pdf]

Copyright WILEY-VCH Verlag GmbH & Co. KGaA, 69469 Weinheim, Germany, 2015.

Supporting Information

for *Adv. Sci.,* DOI: 10.1002/advs.201400020

Phosphorus-Graphene Nanosheet Hybrids as Lithium-Ion Anode with Exceptional High-Temperature Cycling Stability

*Zhaoxin Yu, Jiangxuan Song, Mikhail L. Gordin, Ran Yi, Duihai Tang, and Donghai Wang**

Supporting Information

Phosphorus-Graphene Nanosheet Hybrids as Lithium-ion Anodes with Exceptional High-Temperature Cycling Stability

Zhaoxin Yu,[†] Jiangxaun Song,[†] Mikhail L Gordin, Ran Yi, Duihai Tang, and Donghai Wang*

Department of Mechanical and Nuclear Engineering, The Pennsylvania State University, University Park, PA, 16802, USA.

Email: dwang@ psu.edu

[†] These authors contribute equally.

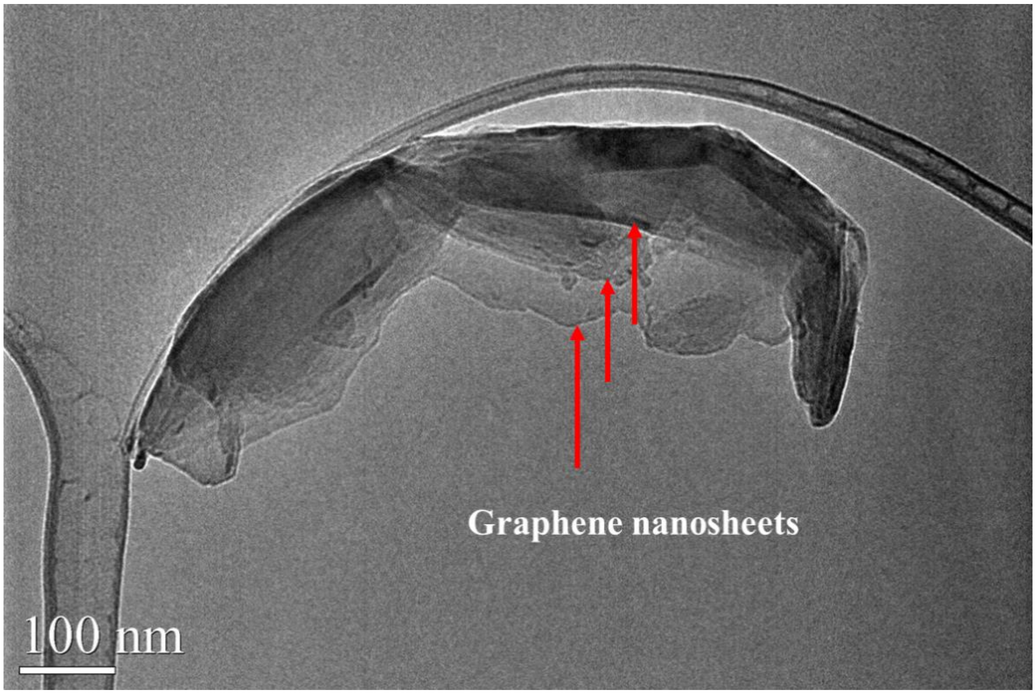

Figure S1 TEM image of graphene nanosheets in the P-G hybrid after removing phosphorus from P-G hybrid by heating the hybrid to 450°C under vacuum.

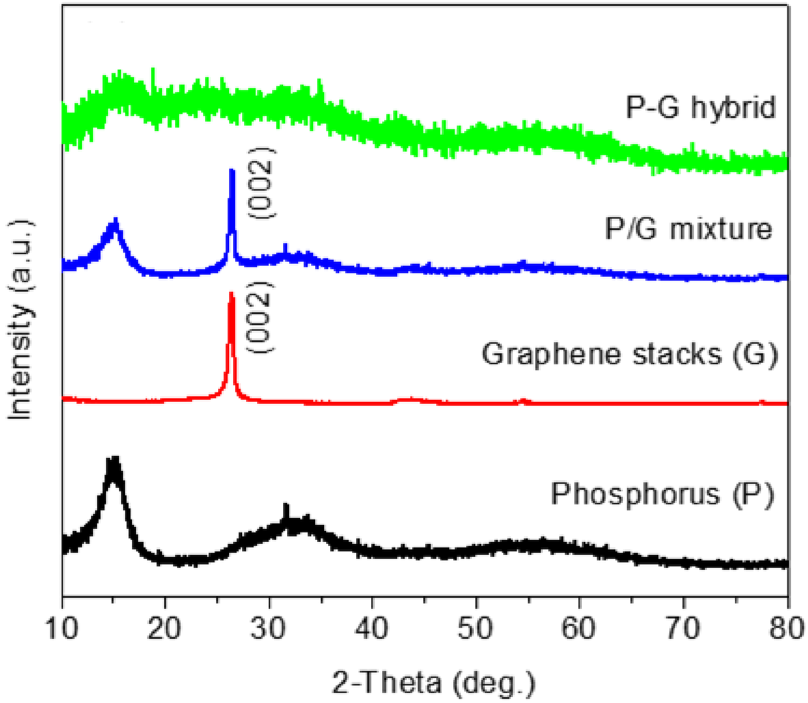

Figure S2 (a) XRD patterns of red phosphorus (P), graphene stacks (G), P/G mixture before ball milling and ball-milled P-G hybrid.

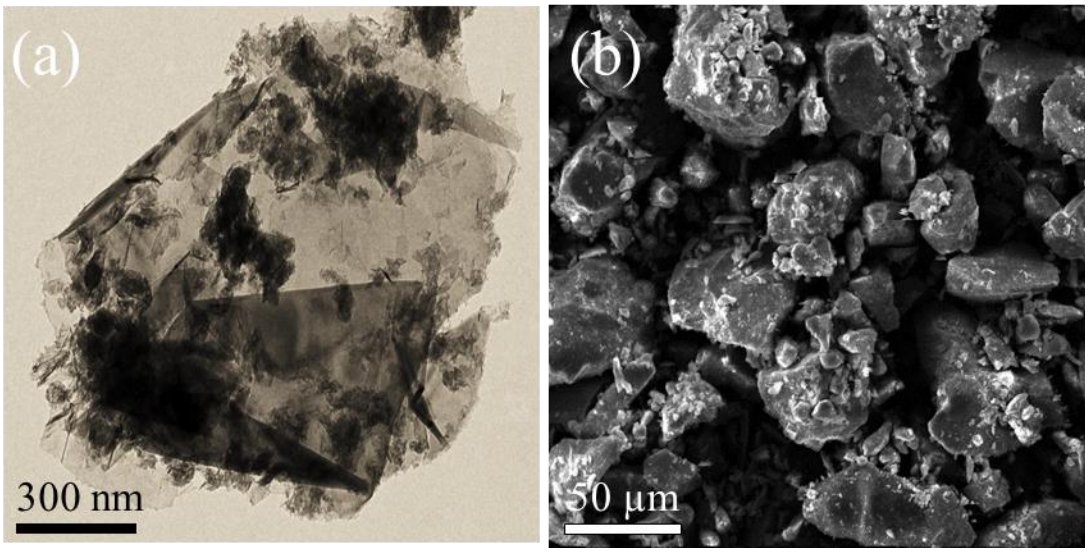

Figure S3 (a) TEM image of graphene stacks and (b) SEM image of raw phosphorus.

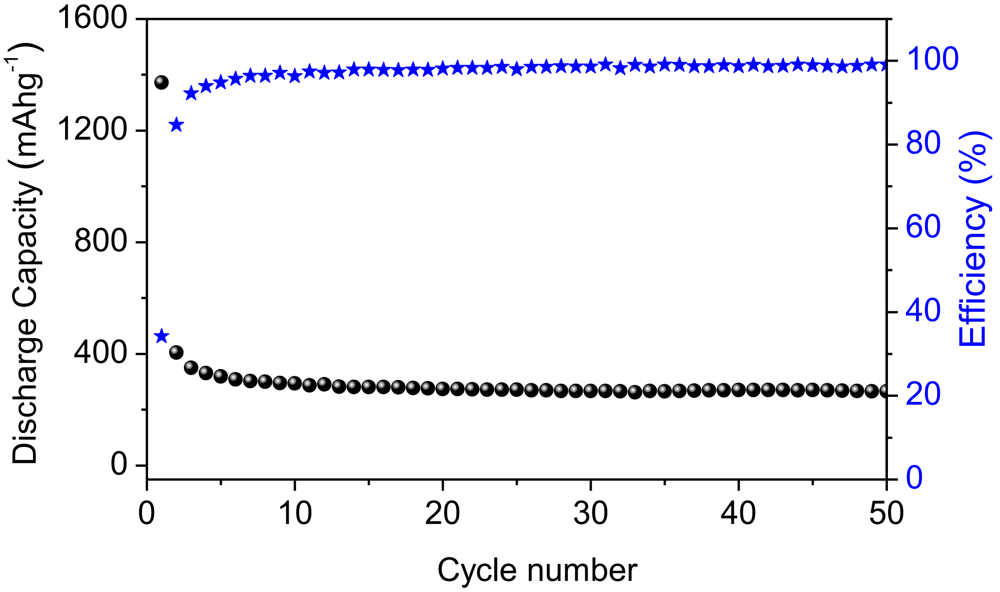

Figure S4 The specific capacity and efficiency of a cell with graphene nanosheets as the active material cycled at a current density of 260 mAg^{-1} at room temperature.

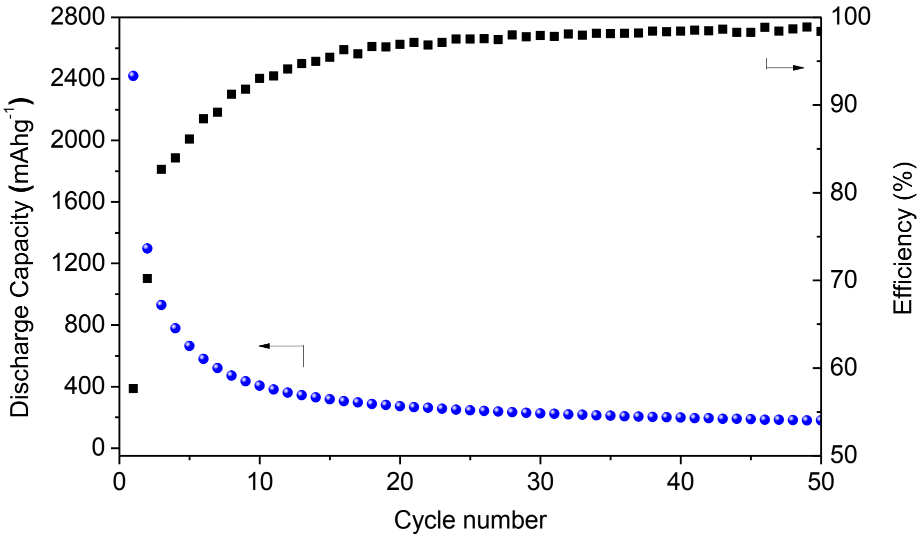

Figure S4 The cycling performance and Couloumbic efficiency of P/G control sample cycled at a current density of 260 mAg^{-1}.
